# Gene Expression in Human Hippocampus from Cocaine Abusers Identifies Genes which Regulate Extracellular Matrix Remodeling

**DOI:** 10.1371/journal.pone.0001187

**Published:** 2007-11-14

**Authors:** Deborah C. Mash, Jarlath ffrench-Mullen, Nikhil Adi, Yujing Qin, Andrew Buck, John Pablo

**Affiliations:** 1 Department of Neurology, Miller School of Medicine, University of Miami, Miami, Florida, United States of America; 2 Department of Cellular and Molecular Pharmacology, Miller School of Medicine, University of Miami, Miami, Florida, United States of America; 3 GeneLogic, Inc., Gaithersburg, Maryland, United States of America; Minnesota State University Mankato, United States of America

## Abstract

The chronic effects of cocaine abuse on brain structure and function are blamed for the inability of most addicts to remain abstinent. Part of the difficulty in preventing relapse is the persisting memory of the intense euphoria or cocaine “rush”. Most abused drugs and alcohol induce neuroplastic changes in brain pathways subserving emotion and cognition. Such changes may account for the consolidation and structural reconfiguration of synaptic connections with exposure to cocaine. Adaptive hippocampal plasticity could be related to specific patterns of gene expression with chronic cocaine abuse. Here, we compare gene expression profiles in the human hippocampus from cocaine addicts and age-matched drug-free control subjects. Cocaine abusers had 151 gene transcripts upregulated, while 91 gene transcripts were downregulated. Topping the list of cocaine-regulated transcripts was RECK in the human hippocampus (FC = 2.0; p<0.05). RECK is a membrane-anchored MMP inhibitor that is implicated in the coordinated regulation of extracellular matrix integrity and angiogenesis. In keeping with elevated RECK expression, active MMP9 protein levels were decreased in the hippocampus from cocaine abusers. Pathway analysis identified other genes regulated by cocaine that code for proteins involved in the remodeling of the cytomatrix and synaptic connections and the inhibition of blood vessel proliferation (PCDH8, LAMB1, ITGB6, CTGF and EphB4). The observed microarray phenotype in the human hippocampus identified RECK and other region-specific genes that may promote long-lasting structural changes with repeated cocaine abuse. Extracellular matrix remodeling in the hippocampus may be a persisting effect of chronic abuse that contributes to the compulsive and relapsing nature of cocaine addiction.

## Introduction

A major goal in drug abuse research is to identify key molecular mechanisms that underlie the development of compulsive drug use. The persisting cravings for cocaine that remain after a protracted period of withdrawal may be due to long-lasting structural changes in certain brain regions. Recent observations suggest that hippocampal learning and memory of the drug euphoria may drive the maladaptive behaviors that increase the risk of relapse to cocaine use [1]–[3]. The hippocampus is involved in short and long-term memory processing [4] and one important target of hippocampal projections is the nucleus accumbens (NAC), a region involved in drug reward circuitry [for review, 5]. Synchronous activity in the hippocampus and nucleus accumbens may be a motivation-to-action interface [6]. Recent behavioral data demonstrate that hippocampal theta stimulation is sufficient to drive reinstatement of cocaine intake in rats extinguished from self-administration [7]. Long-term potentiation (LTP) in the rat hippocampus is modulated by cocaine exposure, suggesting further that drug-induced changes in the hippocampal formation may have some role in the addictive state [8].

By using high density genome-wide arrays, we profiled hippocampal gene expression in cocaine abusers to identify new targets that may play a role in cocaine dependence. Target validation and protein measures were done for selected genes to further confirm functional relevance. This transcriptome survey in the human hippocampus identified an unexpected elevation in RECK (reversion-inducing-cysteine-rich protein with kazal motifs), an endogenous inhibitor of matrix metalloproteinases (MMPs). MMPs remodel the pericellular environment, primarily through cleavage of extracellular matrix (ECM) proteins and receptors [9]–[11]. Brain ECM proteins form the scaffolding for neurons and glia to cling to and make up approximately 20% of the brain. The balance in endogenous tissue inhibitors of MMP activity sustain or break down existing cell adhesion molecules, permitting the reconfiguration of synaptic connections. Gene expression identified hippocampal transcripts involved in angiogenesis, cell adhesion, synaptic formation and cell communication that were regulated by cocaine exposure. Regional gene expression results shown here provide evidence for active transcripts that function to remodel the hippocampal extracellular matrix in human cocaine addiction.

## Results

Brain tissues were taken from autopsy cases according to criteria described by the National Association of Medical Examiners (NAME) Committee on Cocaine-related Deaths for documenting, interpreting and certifying potential cocaine-related fatalities [12], [13]. The investigation of any drug-related death requires knowledge of the circumstances of death, the death scene, and past medical history. It is also necessary to have the results of the forensic toxicologic analysis and those of a forensic autopsy examination prior to classifying that a cause and manner of death is associated with acute cocaine exposure or chronic cocaine use that leads ultimately to a fatal pathologic process. The cocaine users had long-standing histories of cocaine abuse and all subjects had informant reports of “binge” cocaine use in the days immediately before death. All of the cocaine deaths were due to cocaine intoxication. Cocaine and its main metabolite benzoylecgonine (BE) were measured in blood and urine at the time of death for all cases (Table 1). Control subjects were age-matched and drug-free. The manner of death for the control subjects was classified as a natural or accidental death. Three of the control cases were homicide victims of gun shot wounds, one was a blunt trauma death, and the remaining seven cases died from sudden cardiac death. Sudden cardiac death is an event that is non-traumatic, non-violent, unexpected, and resulting from sudden cardiac arrest within six hours of previously witnessed normal health [14]. Review of the cause and manner of death demonstrated that all of the cocaine abusers and control subjects died suddenly without evidence of any significant agonal conditions. Persons that suffered prolonged agonal states, such as with respiratory arrest, multi-organ failure or coma, have lower pH in the brain, while those who experienced brief deaths, associated with accidents or cardiac events have normal pH values [15].

**Table 1:** Demographics, postmortem interval (PMI), brain pH and toxicology

*Group*	*N*	*Age*	*Ethnicity*	*Gender*	*PMI*	*pH*	*Cocaine*	*BE*	*CE*
CTRL	11	31.9±3.5	7C, 4B	10M, 1F	14.4±1.9	6.53±0.04	n.d.	n.d.	n.d.
COC	10	34.0±1.9	7C, 3B	9M, 1F	13.1±1.1	6.50±0.07	6.51±2.69	5.41±1.21	0.44±0.12 (5 out of 10)

**Abbreviations**: BE: benzoylecgonine; CE, cocaethylene; COC, cocaine abuser; CTRL, drug-free control.

### Regulation of Gene Expression by Cocaine

Correlational analysis of postmortem interval (PMI) and RNA quality control parameters showed no significant effect for the two sample groups in the human hippocampus for the initial cohort of cocaine cases (n = 10) and control subjects (n = 11) (Pearson correlation, *R^2^* = 0.045). Analysis of the demographic parameters demonstrated no significant differences in terms of age, PMI or pH between the control subjects and cocaine abusers (Table 1). Individual data for the QC metrics for the total number of cases and controls are shown as supplementary materials (Table S1). Careful Affymetrix QC analysis and matching of the microarrays gave a final cohort of age-matched controls (n = 8) and cocaine abusers (n = 8). Table 2 summarizes the QC metrics for this final cohort of controls and cocaine abusers. We observed no significant correlation between quality control parameters such as noise (RawQ), number of genes detected as present across arrays, scale factor, β-actin and GAPDH 5′/3′ ratios and pH or PMI in several regions tested. These data suggest that RNA quality was acceptable, probably reflecting our brain recruitment procedures, which are limited to sudden death without medical intervention, prolonged agonal periods or extended PMI.

**Table 2:** Quality control parameters for brain sample microarrays from hippocampus.

	*RawQ*		*Scale Factor*	*Present Calls*	*Actin 5*′*/3*′ *ratio*	*GAPDH 5*′*/3*′ *ratio*
**U133A, Mean±SEM**						
**CTRL**	2.77±0.17		0.95±0.12	9646±215	0.51±0.04	0.85±0.03
**COC**	2.71±0.11		0.93±0.11	10081±359	0.42±0.05	0.84±0.07
**U133B, Mean±SEM**						
**CTRL**		2.43±0.14	2.33±0.17	6629±203	0.51±0.04	0.72±0.05
**COC**		2.86±0.18	2.24±0.19	6573±140	0.43±0.05	0.67±0.06

Abbreviations: COC, cocaine abuser; CTRL, drug-free control; GAPDH, glyceraldehyde-3-phosphate dehydrogenase

* The lower percent present calls in the B chip compared to the A chip is due to the fact that the B chip contains primary probe sets representing EST clusters. As a result overall signal intensities on the B chip are lower which is reflected by higher scaling factors. Note that RNA-QC metrics (including β-actin and GAPDH signal ratios) are consistent across chips. Values were derived from Microarray Analysis Suite version 5.0 analysis (available at http://www.affymetrix.com).

**Figure 1:**
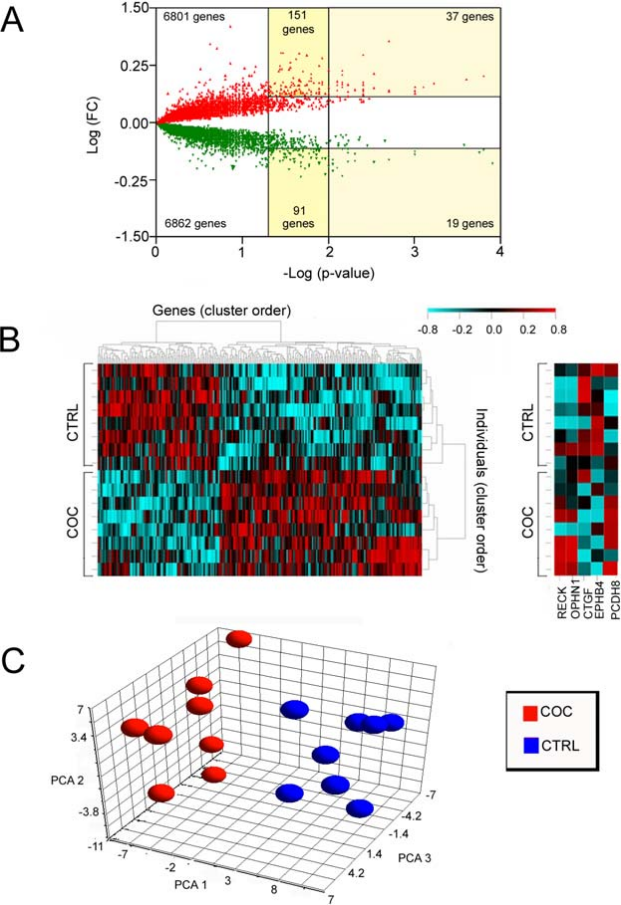
Gene expression in the human hippocampus for cocaine abusers and control subjects. *A,* Volcano plot illustrating the total number of genes (13,662) meeting the criterion (≥75% present call). For all genes detected on the Affymetrix Human Genome HGU133 A & B Chip Set, each point represents a gene plotted as a function of fold-change (Log (fold change), y-axis) and statistical significance (-Log (*p*-value), x-axis). Vertical lines represent *p* values of 0.05 and 0.01, respectively. The upper and lower horizontal lines represent fold changes of +1.3 and −1.3, respectively. Green represents downregulation, while red illustrates upregulation in group comparisons. *B,* Clustered Image Map (left panel) of the relative change in gene expression between the control and cocaine exposure groups. The color change reflects relative change according to the scale shown; red indicates positive fold change and blue indicates negative fold change. B) Representative genes (left panel): RECK, OPHN1, CTGF, EPHB4 and PCDH8 (from left to right). *C,* Principal Components Analysis (PCA) reveals separation between groups for the 242 differentially expressed genes. Red represents the cocaine abusers (COC, n = 8) and blue illustrates the control subjects (CTRL, n = 8).

Using hierarchical clustering, we compared the gene expression profile of this final set of cocaine abusers to that of the normal controls. A Volcano plot illustrates the variance in gene numbers at different p-values (Fig. 1a). Gene expression changes in postmortem brain are usually modest (less than twofold) in studies of psychiatric disorders [16]. A total of 242 differentially expressed genes in the hippocampus of cocaine abusers were observed using combined criteria (±1.3 fold-change and p≤0.05). Of these 242 differentially expressed genes, 151 genes were upregulated and 91 genes were downregulated in cocaine abusers as compared to the age-matched and drug-free control subjects.

Additional confirmation of these selected genes was obtained with hierarchical clustering and shown as a Clustered Image Map (CIM) for expression of the 242 genes between control subjects and cocaine abusers (Fig. 1b). The CIM clearly illustrates the separation of the cocaine abusers from control subjects with a defined cluster order for the individual subjects. Additionally, two distinct gene cluster patterns for the cocaine abusers were observed: a large upregulation and a smaller downregulation pattern of expression. However, the individual genes of interest each showed very clearly defined clusters with primarily unknown genes (EST's and hypothetical proteins). With RECK and protocadherin 8 (PCDH8), the genes were up-regulated by cocaine exposure (Fig. 1b). The genes coding for connective tissue growth factor (CTGF) and EPH receptor B4 (EphB4), were downregulated in this cluster. Results of the principal components analysis (PCA) of the initial 15,336 gene-set resulting from “Present Calls” (MAS 5.0) on the HG-U133 A chip from control subjects and cocaine abusers, demonstrated that the first three components accounted for 35.2% of the total variance (data not shown). PCA analysis based on the initial gene set did not discriminate cocaine abusers from controls. However, PCA analysis based on the selected 242 differentially regulated genes did discriminate groups (Fig. 1c). The first three components accounted for 52.9% of the total variance with components 1, 2 and 3 accounting for 36.3, 9.5 and 7.1% of the variance, respectively.

### Biopathway and cluster analysis

Gene Ontology (GO) analysis using GoSurfer revealed major categories for genes significantly altered by cocaine, which demonstrated changes in gene expression related to specific molecular functions, including angiogenesis, apoptosis and cell death, cell adhesion, neurogenesis and axon guidance. Our brain gene expression was used only as a first step to screen for potential targets dysregulated by cocaine in the human hippocampus in order to identify molecular mechanisms that may be relevant to cocaine dependence. Of particular interest was the observation of a number of different molecular functions associated with remodeling of the cytomatrix. Within the cell adhesion pathway, there were 15 genes differentially regulated by cocaine (Table 3). Topping the list of upregulated transcripts in this category of molecular function was RECK. Other upregulated transcripts associated with cell adhesion and matrix remodeling included laminin beta 1 (LAMB1), integrin beta 6 (ITGB6), three members of the protocadherin family (PCDH8, PCDHA2, PCDHGA1), catenin, beta interacting protein 1 (CTNNBIP1), fibronectin like domain containing leucine rich transmembrane protein 3 (FLRT3), connective tissue growth factor (CTGF) and cell differentiation antigen CD44. There were three transcripts upregulated by cocaine that are associated with neurogenesis and axon guidance pathways, including oligophrenin 1 (OPHN1), axotropin (AXOT), and liprin beta 1 (PPFIBP1). The most commonly overrepresented ontologies-genes based on the rankings and P-values in the hippocampus from the GSR analysis in ErmineJ. were similar to GoSurfer and included genes in the molecular function category (7 classes) and one biological process class (cell cycle). In this analysis, MCM3 and ATP8A2 were the two most over-represented genes, closely followed by one of our validated genes, EphB4.

**Table 3:** Summary and functional categorization of select genes displaying significant fold change (|FC|>1.3) in hippocampus of cocaine abusers.

*Gene Symbol*	*Accession No.*	*Protein Name*	*Fold-Change*	*P-Value* 1	*P-Value* 2
***Angiogenesis***					
RECK	NM_021111.1	Reversion-inducing-cysteine-rich protein with kazal motifs	2.02	0.034	0.0452
MAN1	NM_166651.1	Integral inner nuclear membrane protein	1.46	0.022	0.0415
CCM1	NM_001013406.1	Cerebral cavernous malformations 1	1.44	0.047	0.0471
RSN	NM_002956.2	Restin	1.35	0.010	0.0398
EGFR	NM_005228.3	Epidermal growth factor receptor	−1.30	0.033	0.0427
CSRP2	NM_001321.1	Cysteine and glycine-rich protein 2	−1.36	0.017	0.0402
EPHB4	NM_004444.4	Ephrin type-B receptor 4	−1.47	0.005	0.0380
CTGF	NM_001901.2	Connective Tissue Growth Factor	−1.52	0.017	0.0402
***Apoptosis & Cell Death***					
PPID	NM_001004279.1	Peptidylprolyl isomerase D (cyclophilin D)	1.73	0.035	0.0443
PDCD7	NM_005707.1	Programmed cell death 7	1.38	0.033	0.0415
REQ	NM_006268.3	Requiem, apoptosis response zinc finger gene	1.37	0.002	0.0390
PDCD8	NW_004208.2	Programmed cell death 8	1.35	0.018	0.0402
INHA	NM_002191.2	Inhibin, alpha	−1.32	0.014	0.0402
ABS	NM_016222.2	Dead-box protein abstrakt	−1.42	0.033	0.0426
***Cell Adhesion***					
RECK	NM_021111.1	Reversion-inducing-cysteine-rich protein with kazal motifs	2.02	0.034	0.0452
LAMB1	NM_002291.1	Laminin, beta 1	1.67	0.011	0.0427
SGCB	NM_000232.3	Beta sarcoglycan	1.50	0.013	0.0427
PCDHGA1	NM_018912.2	Protocadherin gamma subfamily alpha, 1	1.48	0.011	0.0398
MAN1	NM_166651.1	Integral inner nuclear membrane protein	1.46	0.022	0.0415
PCDH8	NM_002590.2	Protocadherin 8	1.45	0.043	0.0474
LIN7C	NM_018362.2	Lin-7 homolog C (C. elegans)	1.44	0.032	0.0402
FLRT3	NM_013281.2	Fibronectin leucine rich transmembrane protein 3	1.40	0.024	0.0468
PCDHA2	NM_018905.2	Protocadherin alpha 2	1.36	0.024	0.0402
ITGB6	NM_000888.3	Integrin, beta 6	1.35	0.006	0.0398
RSN	NM_002956.2	Restin	1.35	0.010	0.0398
CTNNBIP1	NM_001012329.1	Catenin, beta interacting protein 1	−1.33	0.023	0.0415
CD44	NM_000610.3	CD44 antigen (homing function)	−1.37	0.014	0.0402
CMAR	NM_003119.2	Cell matrix adhesion regulator	−1.49	0.049	0.0498
CTGF	NM_001901.2	Connective Tissue Growth Factor	−1.52	0.017	0.0402
***Neurogenesis and Axon Guidance***					
PPFIBP1	NM_003622.2	Liprin beta 1	1.52	0.050	0.0481
OPHN1	NM_002547.1	Oligophrenin 1	1.47	0.027	0.0414
AXOT	NM_022826.2	Axotrophin	1.34	0.011	0.0398
SHANK2	NM_012309.1	SH3 and multiple ankyrin repeat domains 2	−1.37	0.005	0.0368
SEMA6A	NM_020796.3	Semaphorin 6A	−1.45	0.009	0.0398
***Receptors & Signal Transduction***					
SGKL	NM_001033578.1	Serum/glucocorticoid regulated kinase-like	1.91	0.024	0.0431
TIPRL	NM_001031800.1	Putative MAPK activating protein	1.33	0.019	0.0427
SYT11	NM_152280.2	Synaptotagamin XI	1.33	0.040	0.0443
CCKBR	NM_176875.2	Cholecystokinin B receptor	−1.30	0.007	0.0398
EGFR	NM_005228.3	Epidermal growth factor receptor	−1.30	0.033	0.0427
PAK6	NM_020168.3	p21 CDKN1A -activated kinase 6	−1.32	0.014	0.0402
DRD2	NM_000795.2	Dopamine Receptor D2	−1.35	0.029	0.0402
SHANK2	NM_012309.1	SH3 and multiple ankyrin repeat domains 2	−1.37	0.005	0.0368
EPHB4	NM_004444.4	Ephrin type-B receptor 4	−1.47	0.005	0.0380
PTPRB	NM_002837.3	Protein tyrosine phosphotase, recceptor type, B	−1.47	0.005	0.0317
PIM1	NM_002648.2	Proto-oncogene serine/threonine-protein kinase	−1.58	0.008	0.0402
***Transcriptional & Translational Regulators***					
NFE2L1	NM_003204.1	Nuclear factor (erythroid-derived 2)-like 1	1.38	0.016	0.0402
RYBP	NM_012234.4	RING1 and YY1 binding protein	1.37	0.027	0.0463
NFYB	NM_006166.3	Nuclear transcription factor Y, beta	1.30	0.055	0.0498
MEF2D	NM_005920.2	Myocyte enhancer factor 2D	−1.38	0.032	0.0443
***Ion Channels & Transport***					
HCN2	NM_001194.2	hyperpolarization activated cyclic nucleotide-gated potassium channel 2	1.78	0.037	0.0459
SLC35A3	NM_012243.1	Solute carrier family 35 (UDP-N-acetylglucosamine (UDP-GlcNAc) transporter), member 3	1.35	0.031	0.0426
CLIC1	NM_001288.4	Chloride intracellular channel 1	−1.35	0.045	0.0464
SLC9A2	NM_003048.3	Solute carrier family 9 (sodium/hydrogen exchanger), isoform 2	−1.35	0.015	0.0398
ORCTL4	NM_004803.2	Organic cationic transporter-like 4	−1.45	0.028	0.0402
KCNAB1	NM_003471.2	Potassium voltage-gated channel, shaker-related subfamily beta member	−1.46	0.014	0.0402

1 *P-values* obtained from ANOVA t-test.

2 *P-values* corrected for multiple testing by the Benjamini-Hochberg method.

A number of different receptors, ion channels and transporters were regulated in the hippocampus by cocaine (Table 3). These included the D2 dopamine (DRD2) and cholecystokinin receptor (CCKBR). The hyperpolarization-activated cyclic nucleotide-gated potassium channel 2 (HCN2) was upregulated 1.8-fold. The KCNAB1 potassium channel gene was downregulated in contrast to the marked upregulation of HCN2. There were six regulators of apoptosis and cell death, four transcripts upregulated and two downregulated by chronic cocaine exposure.

As the final control group selected from the PCA analysis consisted of one female and seven male patients compared with a cocaine cohort of eight males (242 dysregulated genes), we further tested for a possible gender effect between the two groups. For this comparison, the control group had the female control (age 24 yrs.) and one age-matched male cocaine abuser (age 23 yrs.) removed to give a final count of seven subjects per group. There were 250 genes dysregulated by cocaine (163 and 87 up- and down regulated, respectively) as compared to the 242 genes. However, the genes in common for both datasets demonstrated further that there was no apparent gender bias resulting from the inclusion of the one female control subject on the genes of interest (data not shown). Thus, the validation of selected transcripts was done using the same cohort of eight subjects from the cocaine and control groups.

### Target Validation

**Figure 2:**
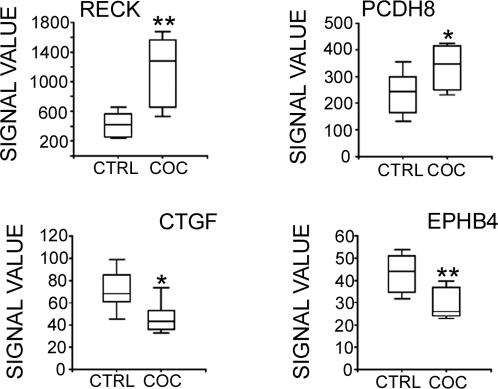
Graphic representation of the observed changes in RECK, PCDH8, CTGF, and EPHB4 gene expression. Raw Affymetrix data (Microarray Analysis Suite version 5.0) illustrate the differential expression of these genes in the hippocampus. Box plot illustrates range and median values. **p≤0.001; *p≤0.01

**Figure 3:**
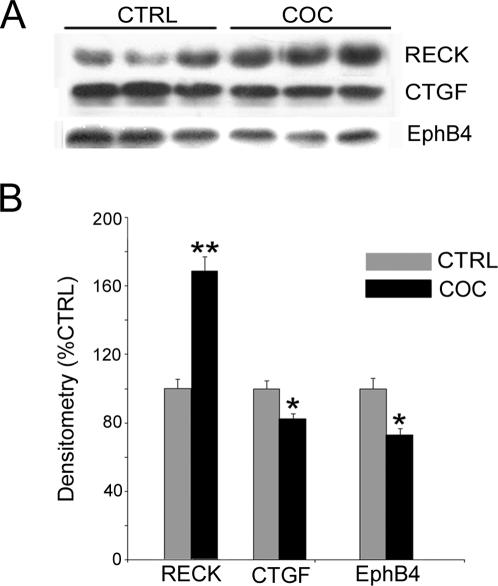
Confirmation of cocaine-regulated transcripts. *A,* RT-PCR confirmation of a sample of four genes (RECK, PCDH8, CTGF, EPHB4) in human hippocampus. Representative cases are shown. All genes were normalized to an endogenous control gene cyclophilin. *B,* Graphical representation of the relative mRNA levels (% of cyclophilin) in control and cocaine groups (n = 8, respectively). **p = 0.01 (one-tailed t-test), *p = 0.05 (one-tailed t-test)

**Figure 4:**
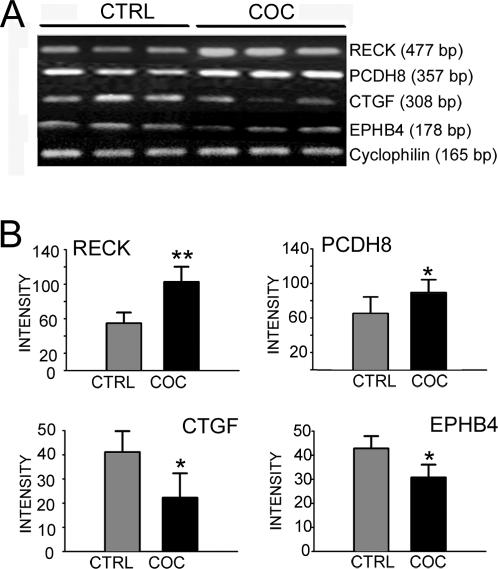
Western blot analysis of RECK, CTGF, and EphB4 protein expression in human postmortem hippocampus. *A,* Representative immunoblots with antibodies against human RECK, CTGF, and EPHB4 demonstrate elevated RECK and decreased CTGF, EPHB4 immunopositive bands in the human hippocampus from cocaine overdose subjects as compared with age-matched, drug-free control subjects (n = 8, respectively). *B,* Quantitation of protein expression. Optical density measurements are shown as percent control (y-axis). Data are means±SEM. **p = 0.01 (one-tailed t-test), *p = 0.05 (one-tailed t-test).

Of the total number of dysregulated genes in the human hippocampus, RECK had the largest upregulated fold-change in cocaine abusers (FC = 2.0; p<0.05). RT-PCR was used to validate the differential mRNA expression levels of RECK and three selected genes that were identified by functional cluster analysis (PCDH8, CTGF and EphB4; Fig. 1b). Differential expression of RECK was confirmed on independent samples from the same cases (Figs. 2 and 3). Altered expression of three other transcripts PCDH8, CTGF and EphB4 were confirmed by RT-PCR analysis (Fig. 3) in agreement with the microarray results shown in Figure 2. Confirmation of protein levels for three of the four genes selected was done based on availability of commercial antibodies. Quantitation of immunopositive bands from Western blots demonstrated higher RECK and PCDH8 and lower CTGF protein expression in cocaine abusers compared to age-matched and drug-free control subjects (Fig. 4). Additional experiments were performed on the cohort of cocaine abusers (N = 8) and control subjects (N = 8) to further validate selected gene transcripts in cocaine-induced changes in hippocampal function. The expanded qPCR results of ten genes (RECK, SGKL, HCN2, LAMB1, OPHN1, PCDH8, EphB4, ITGB6, CTGF, CTNNBIP1) normalized to 18S rRNA are shown in Table 4. The fold change values determined by qPCR are in good agreement with the microarray data shown in Table 3 for these transcripts.

**Table 4:** Comparison of relative expression data derived by qPCR analysis.

*Gene* a	*Fold-Change* b	*P-Value*
RECK	1.98	0.015
SGKL	1.95	0.013
HCN2	1.79	0.031
LAMB1	1.73	0.007
OPHN1	1.61	0.035
PCDH8	1.44	0.001
EPHB4	−1.43	0.033
ITGB6	1.35	0.021
CTGF	−1.35	0.002
CTNNBIP1	−1.32	0.047

a For gene names, refer to Table 3

b Negative fold changes correspond to down-regulation of gene expression in cocaine tissue relative to control tissue.

### Downregulation of MMP9 protein in cocaine abusers

**Figure 5:**
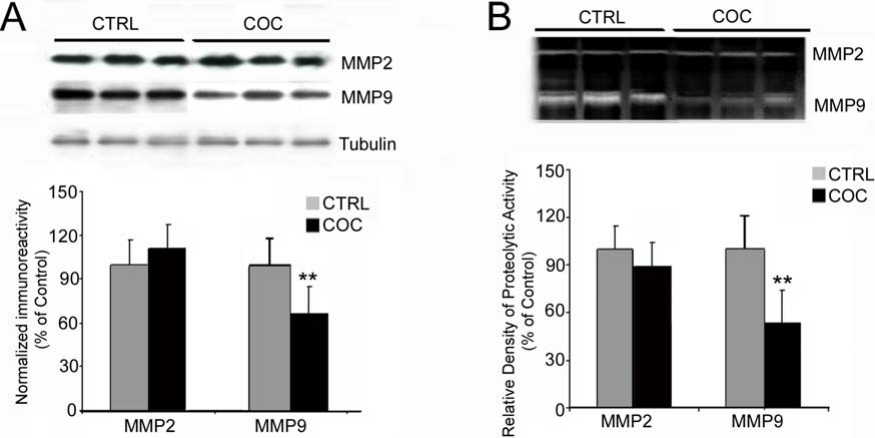
Regulation of MMP9 in human hippocampus with cocaine exposure. *A,* Representative Western immunoblots with antibodies against human MMP2 and MMP9 demonstrate down-regulation of MMP9 protein expression in cocaine abusers compared to age-matched controls subjects (n = 8, respectively). The relative optical densities for active 92 kDa MMP9 and 72 kDa MMP2 illustrate quantitative fold change of MMP2 and MMP9 protein expression. Equal amount of total protein loading was confirmed by α-tubulin. *B*, Representative gel zymography. MMP9 activity in cocaine abusers compared with age-matched drug-free control subjects demonstrate decreased activity. The graphs illustrate quantitative zymography of MMP2 and MMP9. Levels of MMP2 activity were unchanged. Data are means±SEM. **p = 0.01 (one-tailed t-test).

We examined whether the target molecules of RECK, MMP2 and MMP9 were regulated by cocaine abuse. The microarray and RT-PCR results demonstrated that neither transcript was regulated by cocaine in the human hippocampus (data not shown). Previous studies have demonstrated that increased expression of RECK results in downregulation of MMPs at a post-transcriptional level [17], [18]. Since MMP2 and MMP9 are expressed in brain, we conducted additional experiments using Western blot and gelatin zymography to measure protein expression. MMP9 protein in the hippocampus from cocaine users (N = 8) and control subjects (N = 8) gave a single band at the expected molecular mass of 92 kDa (Fig. 5a). MMP9-positive bands were consistently less dense in the cocaine users (p<0.01). A single band was observed for MMP2 at the expected molecular mass of 72 kDa in cocaine users and in control subjects (Fig. 5a). In contrast to MMP9, there was no change in MMP2 and α-tubulin (50 kDa) levels between groups. Densitometric analysis of MMP2 immunoblots gave values for cocaine users that were not different from control subjects. These results demonstrate that the cocaine-induced down-regulation of MMP9 was not accompanied by changes in MMP2.

Representative zymography gels of active MMP2 and MMP9 are shown in Figure 5b. In the human hippocampus, gelatinolytic activities at 72 and 92 kDa corresponded to the proenzyme forms of MMP2 and MMP9, respectively. The zymograms showed a reproducible reduction in MMP9, but no change in MMP2 in cocaine abusers. In keeping with the immunoblot evidence of a decrease in total protein, gelatin zymography demonstrates that cocaine exposure downregulates active MMP9 in human hippocampus. These observations suggest that MMP9 is the molecular target of RECK in the human hippocampus of cocaine abusers.

### Biological Pathway Analysis

**Figure 6:**
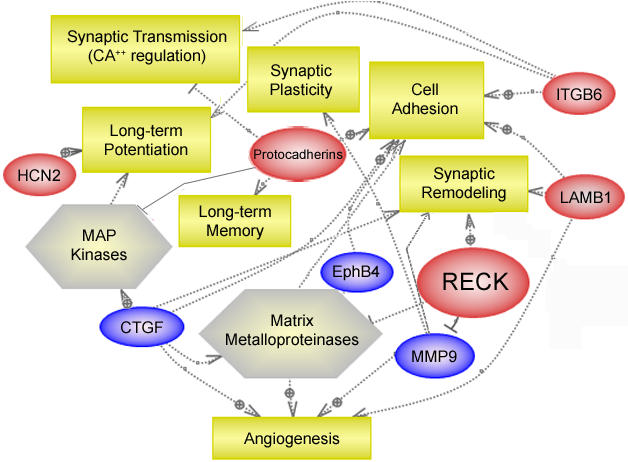
Schematic representation of coordinated gene expression changes and signaling pathways activated in the hippocampus of cocaine abusers compared to age-matched and drug-free control subjects. The biological network was identified by incorporating the gene expression results into Pathway Studio (Ariadne Genomics, Rockville, MD). Oval-shaped symbols represent regulated transcripts, square symbols illustrate cellular or molecular functions and arrows denote some of their possible interactions. Upregulated transcripts are shown in red; purple illustrates downregulated genes. Matrix metalloproteinases (hexagonal symbol, center) are zinc-dependent endopeptidases that degrade numerous extracellular matrix proteins. MAP kinases and other essential kinases play roles in the expression of long-term potentiation. Many of the dysregulated genes associated with cell adhesion and extracellular matrix remodeling are involved in memory and learning processes. Changes in MMP9 activity are a requirement for remodeling of extracellular matrix cell adhesion molecules occurring with synaptic plasticity, which in turn is necessary for memory consolidation. Abbreviations: HCN-2, hyperpolarization activated cyclic nucleotide-gated potassium channel 2; CTGF, Connective Tissue Growth Factor; RECK, Reversion-inducing-cycteine-rich protein with kazal motifs; MMP9, matrix metalloproteinase 9; EphB4, ephrin receptor B4; LAMB1, laminin beta 1; ITGB6, Integrin, beta 6.

The expression array data set allows the assessment of the biological interaction of the protein products using Ariadne Genomics PathwayAssist™ biological pathway visualization and analysis software. Figure 6 illustrates a schematic representation of a potential signaling pathway involving RECK and MMP9 regulation of the cytomatrix in cocaine abusers compared to control subjects. PathwayAssist analysis identified increased expression of members of the protocadherin family and other genes shown in the biological analysis network (BAN) which encode proteins that are part of a signaling pathway for cell adhesion, extracellular matrix remodeling and angiogenesis (ITGB6, LAMB1, CTGF, EphB4). MMPs in the adult brain play a role in remodeling of synaptic connections, a mechanism important for synaptic plasticity, learning and memory. The results of this analysis suggest a coordinated regulation of hippocampal RECK, MMP9, protocadherins and other proteins of the cytomatrix that have not been demonstrated previously in human postmortem studies of gene expression in chronic cocaine abusers.

## Discussion

The present microarray study showed that 242 transcripts were altered in the hippocampus from cocaine abusers compared to control subjects. Increased gene expression in the hippocampus predominated in the cocaine abusers, consistent with an acute state of activation and recent “crack” cocaine use. In keeping with this observation, transcriptional profiling in the human prefrontal cortex provided evidence for two activational states, one that was associated with recency of cocaine abuse [19]. While it is not possible to assign a precise time course for cocaine-induced transcriptional regulation from postmortem studies, surges or peaks in transcriptional activity might occur during states of cocaine craving or euphoria reported by cocaine abusers [20], [21]. The limited number of genes upregulated in the hippocampus of human cocaine abusers suggests that gene induction may be a favored process for long-term neuronal adaptations. The persistent effects of cocaine abuse on brain structure and function likely underlie the inability of most cocaine addicts to remain abstinent. In fact, part of the difficulty in preventing relapse is the intense memory of the drug “high”. Evidence from both animal and human studies of cocaine addiction implicate memory-associated brain regions and processes [21]. Transcriptional responses to reinforcing effects of cocaine in the rat hippocampus demonstrate a similar number albeit different gene profile in cocaine conditioning [22]. The results shown here identify novel gene targets for hippocampal synaptic plasticity in the human that are dysregulated by cocaine exposure.

Matrix metalloproteinases (MMPs) are a large family of extracellularly acting endopeptidases, the canonical substrates of which are proteins of the extracellular matrix and adhesion proteins [23]. In the brain, MMPs and their natural inhibitors (TIMPs and RECK) are critical regulators of the structural and functional remodeling of cellular architecture in the context of pathophysiology [24]. However, functional and structural synaptic remodeling through the MMP pathway is not limited to injury, but is also a component of normal physiology, including the formation of new networks that subserve learning and memory [25]. We observed a significant cluster of gene transcripts associated with synaptic structure and function, including cell-surface adhesion proteins as well as proteins of the extracellular matrix with which they interact. An unexpected finding was the upregulation of RECK in the human hippocampus together with other receptors and proteins which transduce cell-matrix interactions.

MMP9 may regulate synaptic plasticity by enabling structural synaptic remodeling similar to that associated with activity-dependent plasticity [26], [27]. Recent studies in MMP9 knock-out mice provide evidence that MMP9 is regulated with changes in synaptic efficacy that enable late phase LTP and memory [28], [29]. A coordinated regulation of RECK and MMP9 may be part of a signaling cascade that leads to anchoring of adhesion target substrates with dendritic spine remodeling [30]. Previous work has also implicated MMP9 in forms of protracted hippocampal remodeling accompanying epileptogenesis [31]. In humans, a kindling-like progression with cocaine abuse has been linked to the occurrence of panic attacks [for review, 32]. Although none of the cocaine cases used in this study had evidence of a terminal seizure, kindling due to long-term cocaine abuse may lead to adaptive and microstructural changes. At the level of gene expression, the balance of these pathological and adaptive processes probably determines whether a seizure will be manifest or suppressed with chronic cocaine abuse [32].

The cadherin family of synaptic adhesion proteins is associated with structural remodeling during late phase LTP [33]. Gene expression profiling identified cell adhesion-related transcripts, including upregulation of three members of the protocadherin family and ITGB6 in the hippocampus of cocaine abusers. Protocadherins are known targets for MMP-dependent cleavage [34]. MMP9 binds also to synaptic integrins that anchor or position them to cleave other target substrates to modulate synaptic plasticity [35]. The effect of RECK as a modulator of the extracellular milieu is a function of deregulated MMP activities and perturbation of integrin signaling. Integrins are a large family of heterodimeric transmembrane glycoproteins that attach cells to extracellular matrix proteins of the basement membrane or to ligands on other cells [36]. Integrins also modulate fast excitatory transmission at hippocampal synapses [37]. Integrin activation and signaling occurring over several minutes after LTP induction are necessary for stabilizing synaptic potentiation, which may be required for the conversion of new memories into a not readily disrupted state [38]. Cleavage of the extracellular matrix molecule laminin by the plasmin system has been suggested to be important for CA1 LTP [39]. By inference, genes that encode or regulate cell adhesion proteins in the human hippocampus make them likely targets for processes that contribute to the reconsolidation of the memory of the cocaine high.

RECK has been shown in studies of wound healing and cancer metastasis to be a key regulator of the extracellular matrix integrity and an inhibitor of angiogenesis [11], [17]. In the embryonic brain, RECK is specifically expressed in Nestin-positive neural precursor cells [40]. However, the function of RECK in the adult brain in mediating synaptic plasticity in the mature hippocampus is unknown. Although RECK has been shown to negatively regulate both MMP2 and MMP9, we observed a selective change in active MMP9 protein expression in cocaine abusers. RECK inhibits angiogenesis and a deficiency in RECK leads to excessive degredation of the extracellular matrix [17], [41]. Thus, the marked increase in RECK message and protein levels suggests that there may be a build up or enhanced maintenance of a remodeled perivascular space in the hippocampus with chronic cocaine abuse. Extracellular matrices consist mainly of collagen, proteoglycans, and glycoproteins such as laminin and fibronectin [23]. RECK mediated inhibition of angiogenesis would result in fewer vascular capillary sprouts from pre-existing blood vessels to allow for the expansion and rearrangement of the perivascular space. We observed a down regulation of CTGF and EphB4 receptor, two gene transcripts that are regulators of postnatal angiogenesis [42], [43]. Since MMPs can degrade the entire extracellular matrix, coordinated regulation of RECK, MMP9, CTGF and EphB4 suggests expansion of the extracellular matrix occurs with morphological remodeling of the hippocampus in response to chronic cocaine abuse.

The gene expression results demonstrate an upregulation of HCN2 in cocaine abusers that may reflect the expansion and remodeling of hippocampal circuits. Hyperpolarization-activated cyclic nucleotide-gated ion channels (h-channels; HCN) modulate the intrinsic excitability of pyramidal neurons [44], [45]. The hyperpolarization-activated cation channels of the HCN gene family contribute to spontaneous rhythmic activity in both brain and heart ([46], [47]. Increasing evidence implicates h-channels in activity dependent learning and memory [48], [49]. Hippocampal mossy fiber LTP is mediated by presynaptic h-channels [50]. MAP kinases and other essential kinases play a role in late LTP [51]. HCN channels support pacemaking in various excitable cells [52]–[54]. In the globus pallidus, HCN channels determine the rate and regularity of autonomous pacemaking and sculpt the responses to GABAergic input, allowing a persistent pacemaker reset to create synchronous activity [55]. This mechanism may be relevant to cocaine abuse in the context of extracellular matrix remodeling, in that a cocaine induced reset of pacemaker activity might be an underlying activity dependent metaplasticity that functions as a trigger for the consolidation or adaptive generalization of memories of an intense euphoria. Further studies are needed to determine the functional relevance of upregulated HCN channels as a neuroplastic response to cocaine in hippocampal synapses.

A recent single nucleotide polymorphism (SNP) whole genome association study of drug abusers and matched control subjects identified many cell adhesion genes [56]. These included members of the protocadherin family and genes whose products bind to cell adhesion related protein complexes [61], [62]. While these observations do not negate other genes that confer vulnerability to addiction, they do agree with the observations shown here for hippocampal gene expression in cocaine abusers. DRD2 has been shown to be a vulnerability locus for addiction [57], [58]. and PET imaging demonstrates a decrease in DRD2 occupancy in drug addicted subjects [59]. In agreement with these findings, gene expression profiling of the human hippocampus demonstrated the expected decrease in DRD2 expression in cocaine abusers compared to control subjects. However, the results of whole genome SNP analysis together with the gene expression profiling reported here provide further support for a role of memory-associated brain regions in cocaine addiction. Extracellular matrix remodeling in the human hippocampus may be a persisting structural effect of chronic abuse that consolidates the maladaptive memories of a drug-induced euphoria. These cocaine induced changes in hippocampal structure and function may be an underlying neural deficit that makes abstinent cocaine addicts vulnerable to relapse despite the negative consequences of continuing their cocaine habit.

## Methods

### Subjects

Postmortem neuropathological specimens were obtained during routine autopsy from cocaine-related deaths and age-matched drug-free control subjects (Table 1). Medico-legal investigations of the cause and manner of death were conducted by forensic pathologists [12], [13]. The circumstances of death and toxicology data were reviewed carefully before classifying a cocaine intoxication case. Cocaine cases (N = 10) were evaluated for common drugs of abuse and alcohol and positive urine screens were confirmed by quantitative analysis of blood. Blood cocaine and benzoylecgonine were quantified using gas-liquid chromatography with a nitrogen detector. Drug-free age-matched control subjects (N = 11) were selected from accidental or cardiac sudden deaths with negative urine screens for all common drugs and there was no history of licit or illicit drug use prior to death. All subjects died suddenly without a prolonged agonal state. Since agonal state may affect the RNA expression profile of postmortem brain tissue, care was taken to match subject groups as closely as possible for age, gender, PMI, and brain pH. Regional samples of postmortem brain were taken from frozen coronal blocks based on surface and cytoarchitectural landmarks. Hippocampal specimens were collected with postmortem intervals less than 24 hours at autopsy (Table 1). The hippocampus was sampled bilaterally for RNA and protein from coronal slices taken at the anterior level of the hippocampal body, including the dentate gyrus and the Cornu Ammonis fields CA1- CA4 and the subiculum.

### Microarray experiments

Total RNA isolation and biotin-labeled cRNA synthesis were performed by Gene Logic Inc. (Gaithersburg, MD) using a TriZol method and RNEasy columns, according to Affymetrix (Santa Clara, CA) specifications from 50 mg of each regional sample. Mean (SEM) values for postmortem RNA extraction were consistent with excellent preservation of RNA quality, with a A260/A280 ratio of 2.18±0.06 and RIN value of 7.61±0.14 (Agilent Bioanalizer 2100 RNA Integrity Number, Technologies, Palo Alto, CA). We used the Human Genome U133 AB set, containing around 45,000 probesets representing >39,000 transcripts derived from around 33,000 well substantiated human genes (http://www.affymetrix.com). Gene chip analysis was performed with Microarray Analysis Suite version 5.0, Data Mining Tool 2.0, and Microarray database software (available at: http://www.affymetrix.com). The genes represented on the gene chip were globally normalized and scaled to a signal intensity of 100. Expression data was analyzed using Genesis 2.0 (GeneLogic Inc, Gaithersburg, MD) and AVADIS (Strand Genomics, Redwood City, CA). Several RNA integrity measures were used in this study to detect samples with poor RNA quality before final analysis. Microarray quality control parameters used included the following: consistent β-actin and glyceraldehyde-3-phosphate dehydrogenase 5′/3′ (GAPDH) signal ratios, consistent number of genes detected as present across arrays, noise (RawQ) and consistent scale factors to select cases and controls for inclusion in the final microarray analysis (Table 2). Problematic arrays were also detected using principal components analysis (PCA). No significant differences were observed between the two groups in terms of age (p = 0.10) or post-mortem interval (p = 0.13). Consistent with our previous reports, analysis of postmortem interval on RNA quality control parameters revealed no significant effects [60]–[62].

### Data Analysis

Gene analysis was based on ‘present’ calls determined by Microarray Analysis Suite 5.0 and genes were included if detected in at least 75% of the subjects in each group to reduce the number of false positives. Expression data were analyzed using Genesis (GeneLogic, Inc., Gaithersburg, MD) and Avadis software (Strand Genomics, Redwood City, CA). Gene expression values were floored to 1 and then log_2_-transformed. Statistically differentiated genes (control – cocaine comparison) were first identified with the t-test to identify statistically significant fold changes p≤0.05 and a fold change/*P*-value combination of at least a 1.3 fold change (FC) in either direction. The resulting *P*-values were adjusted for multiple testing using the Benjamini-Hochberg procedure for controlling the false discovery rate (Bioconductor Suite v. 2.12.0). Cluster analysis was performed using an average-linkage hierarchical cluster group with a correlation metric. Expression patterns in individual subjects and genes were clustered based on the initial gene sets according to selected criteria.

### Functional Cluster and Automated Pathway Analysis

The Affymetrix probeset identifiers were analyzed with gene ontology (GO) terms and the results were visualized as a hierarchical tree using GoSurfer (http://biosun1.Harvard.edu/complab/gosurfer/). GoSurfer was used to identify GO categories regulated by cocaine exposure by inclusion of lists of differentially expressed genes. Functional ontological profiling of the expression changes was performed using the Gene Score Resampling (GSR) method (ErmineJ software v. 2.1.15, Columbia University, NY) with distributions of scores (FC or P-values) determined across the whole array [63]. The parameters used were the following: maximum gene set size: 200; minimum gene set size: 5; with the mean of replicates, 10,000 iterations and full resampling. The rank and *P*-value computed by ErmineJ were used to calculate the most overrepresented genes. Further functional profiling was then performed using Pathway Studio (version 4.0 Ariadne Genomics, Rockville, MD). The gene list that was generated by the microarray data was imported into Pathway Studio to identify and group genes into specific biopathways. This software uses a proprietary database containing over 140,000 references on protein interactions obtained from PubMed to generate a biological association network (BAN) of known protein interactions. By overlaying microarray expression data onto the BAN, co-regulated genes that define a specific signaling pathway were identified to graphically illustrate all known relationships between differentially expressed genes. The list of differentially expressed genes was viewed also by visual inspection manually to confirm the output of functional profiling tools.

### Target validation and Protein Studies

Regional samples from all subjects included in the final microarray analysis were used to validate positive findings for selected transcripts by reverse transcription PCR (RT-PCR) and quantitative real-time PCR and to confirm their relevance at the protein level (Western blot and gel zymography). Subjects were selected with researchers blinded to the microarray results.

### RT-PCR reagents and cycling

RNA was extracted by TRIzol reagent (Invitrogen Life Technologies, Carlsbad, CA) followed by cDNA synthesis. For each sample, 2 µg RNA was used for reverse transcription reaction with SuperScript First-Strand Synthesis system (Invitrogen Life Technologies, Carlsbad, CA). cDNA synthesis was carried out in a total volume of 20 µl. PCR was performed using PCR Master Mix (Promega, Madison WI) in an Amplitron II (Thermolyne, Dubuque, IA. USA). The PCR cycle consisted of denaturation for 1 minute at 95°C, annealing for 1 minute at 56°C, and extension for 2 minutes at 72°C followed by 5 minutes at 72°C. Standard curves were constructed for each RT-PCR assay. Cyclophilin was used as an internal standard. The primer pairs used to assess expression levels were as follows: RECK (30 cycles): 5′-CCTCAGTGAGCACAGTTCAGA-3′, and 5′-GCAGCACACACACTGCTGTA-3′, CTGF (28 cycles) 5′-GAATGTAAA GCTTGTCTGATCG-3′ and 5′-CATGTAACTTTTGGTCACACTC-3′, PCDH8 (26 cycles) 5′-TCTGGCAGAGAAGCAGAGAAG-3′ and 5′-GTGCA GTACTTTCTCATAGAC-3′, EphB4 (32 cycles) 5′-GTCTGACTTTGGCCTTTCCC-3′ and 5′-TGACATCACCTCCCACATCA-3′, and cyclophilin (22 cycles) 5′-TCCTAAAGCATA CGGG TCCTGGCAT-3′, and 5′-CGCTCCATGGCC TCCACAATATTCA-3′. The PCR products were visualized by 1.5% agarose gel electrophoresis. Images of gels were evaluated for differences in band size and intensity.

### Real-time PCR

Gene expression of selected target genes was measured in each sample by real-time PCR using TaqMan Universal PCR Master Mix and the Applied Biosystems 7900HT thermocycler (ABI, Foster City, CA). TaqMan probes and proprietary primers were designed based on previously reported sequences were purchased from ABI (Foster City, CA).

The concentration of RNA was determined by spectrophotometry, using Nanodrop-1000 (Nanodrop Technologies, Wilmington, DE). Reverse transcription was performed with High-Capacity cDNA Reverse Transcription kit using random primers from ABI (Foster City, CA). Gene expression levels were normalized to those of the internal reference 18S rRNA. All samples were run in duplicate reactions and no template control runs were performed for each primer pair. cDNA was amplified using TaqMan Universal PCR master mix reagent (ABI, Foster City, CA) at the following conditions: 2 minutes at 50°C, 10 minutes at 95°C, 40 cycles: 15 seconds at 95°C and 1 minute at 60°C. The target cDNA for RECK, SGKL, LAMB1, PCDH8, ITGB6, HCN2, CTGF, OPHN1, CTNNBIP1, EPHB4 was amplified using TaqMan ABI MGB probe and primer set assay; Hs00221638_m1, Hs00179430_m1, Hs00158620_m1, Hs00159910_m1, Hs00168458_m1, Hs00606903_m1, Hs00170014_m1, Hs00609994_m1, Hs00172016_m1, Hs00174752_m1 respectively and normalized to 18S rRNA as a control (ABI MGB probe and primer set assay ID Hs99999901_s1). Levels of 18S rRNA did not differ between the control and cocaine groups. Normalized Ct values (calculated automatically within the Applied Biosystems software RQ manager 1.2.) were used to calculate expression ratios between the cocaine and control groups. Expression ratios were subjected to a log2 transform to produce fold change data. Student's t-test was used to test for significant differences between control and cocaine groups. One-way analysis of variance (ANOVA) was used to compare gene expression with a Tukey's post-hoc comparison (SPSS, Inc.).

### Western Blot Analysis

Brain samples from hippocampus (100 mg tissue punch) were homogenized with 35 strokes of a pestle in a glass homogenizer in 10 volumes of ice-cold homogenization buffer containing protease inhibitors (50 mmol/L Tris.Cl, pH 7.4, 150 mmol/L NaCl, 1% Nonidat P-40, 0.1% SDS and 0.1% deoxycholic acid with 1×Protease Inhibitor Cocktail, Sigma, St. Louis, MO) and centrifuged at 12,500×g for 30 min at 4°C. The supernatants were collected and the total proteins were assayed using the bicinchoninic acid assay kit (Pierce Chemical, Rockford, IL). Protein extracts were processed on SDS-PAGE on 8% separating and 4% stacking gel and transferred to Immobilon-P nitrocellulose membrane (Millipore, Bedford, MA). The membranes were blocked with 3% BSA in TBS for 1 hr at room temperature and then incubated overnight at 4°C with the following primary antibodies: anti-RECK (1∶250, BD Transduction Laboratories, San Jose, CA), anti-EphB4 Receptor (1∶200, Zymed Laboratories Inc., South San Francisco, CA), anti-CTGF (1∶5,000, Cell Sciences, Norwood, MA), anti-MMP9 (1∶5000, Chemicon International, Temecula, CA) or anti-MMP2 (1∶5000, Chemicon International). Total protein loading was determined by western blotting to anti-α-tubulin (1∶10,000, Sigma). The membranes were then incubated with horseradish peroxidase-conjugated anti-rabbit or anti-mouse secondary antibody for 1 hour at room temperature (1: 50,000, Pierce Chemical, Rockford, IL). Proteins in the blots were visualized by SuperSignal West Pico Chemiluminescent Substrate (Pierce Chemical). Exposures with maximal signal yet below the photographic saturation point were quantitatively analyzed by densitometry. Optical densities were determined using ImageJ (version 1.36b, NIH Shareware) and expressed as arbitrary units.

### Zymography

Gelatinolytic MMP2 and MMP9 activities were measured in postmortem brain tissue samples by the ultrasensitive gel zymography technique [64]. Equal amounts of protein from each sample homogenate were mixed with sample buffer (62.5 mM Tris·Cl, 2% SDS, 25% glycerol, 0.01% bromophenol blue) and subjected to 10% gelatin Ready Zymogram Gel (Bio-Rad Laboratories, Hercules, CA). Gels were washed twice for 30 min in 2% Triton X-100 and incubated at 37°C for 20 hr in incubation buffer (50 mM Tris·Cl, pH 7.5, 5 mM CaCl_2_, 1 µM ZnCl_2_, 0.01% sodium azide). After incubation, gels were stained in 0.5% Coomassie brilliant blue R-250 with 20% methanol and 10% acetic acid for 90 min. After staining, gels were destained in 35% ethanol and 10% acetic acid for 60 min. MMP2 and MMP9 activity was visible as clear bands on a blue background. Standard protein markers (Bio-Rad Laboratories) and human recombinant MMP2 and MMP9 proteins (Chemicon International) were used to size and confirm the identity of the gelatinolytic bands on the gels.

## Supporting Information

Table S1 Supplemental data table
(0.14 MB DOC)
