# Supplementary material for: Gene Expression in Human Hippocampus from Cocaine Abusers Identifies Genes which Regulate Extracellular Matrix Remodeling
Source: PLoS One. 2007 Nov 14;2(11):e1187. doi: 10.1371/journal.pone.0001187 (PMC2063513; doi:10.1371/journal.pone.0001187)
Supplement: Table S1 — Supplemental data table (0.14 MB DOC) [file pone.0001187.s001.doc]

**Supplemental Table S1. Demographic and quality control metrics for individual hippocampal sample microarrays.**

|  | **Gender** |  | **PMI**  **(hr.)** | **Race** | **Age (yr)** | **U133A**  **β-Actin Ratio** | | **U133B**  **β-Actin Ratio** | | **U133A**  **GAPDH Ratio** | | **U133B**  **GAPDH Ratio** | | **U133A**  **Present Calls** | | **U133B**  **Present Calls** | **U133A**  **RAWQ** | | **U133B**  **RAWQ** | | | **U133A**  **Scale Factor** | | **U133B**  **Scale Factor** |
| --- | --- | --- | --- | --- | --- | --- | --- | --- | --- | --- | --- | --- | --- | --- | --- | --- | --- | --- | --- | --- | --- | --- | --- | --- |
| ***Controls*** |  |  |  |  |  |  | |  | |  | |  | |  | |  |  | |  | | |  | |  |
| **1** | M | CTRL | 3.0 | C | **53** | 0.51 | | 0.52 | | 0.79 | | 0.6 | | 8897 | | 5959 | 1.99 | | 2.38 | | | 1.77 | | 2.86 |
| **2** | M | CTRL | 24.0 | B | 39 | 0.46 | | 0.43 | | 0.79 | | 0.61 | | 9558 | | 6905 | 2.4 | | 2.01 | | | 1.19 | | 2.57 |
| **3** | M | CTRL | 18.0 | C | 29 | 0.77 | | 0.7 | | 0.99 | | 0.99 | | 9344 | | 6680 | 2.61 | | 2.83 | | | 0.96 | | 1.89 |
| **4** | M | CTRL | 9.0 | B | 20 | 0.57 | | 0.58 | | 0.78 | | 0.66 | | 9046 | | 6409 | 2.43 | | 1.98 | | | 1.46 | | 3.03 |
| **5** | M | CTRL | 22.0 | B | 22 | 0.36 | | 0.38 | | 0.75 | | 0.62 | | 9761 | | 7050 | 2.09 | | 2.18 | | | 0.78 | | 1.77 |
| **6** | M | CTRL | 11.5 | C | 34 | 0.48 | | 0.45 | | 0.93 | | 0.79 | | 10609 | | 6664 | 3.44 | | 2.27 | | | 0.51 | | 2.62 |
| **7** | M | CTRL | 12.0 | C | 26 | 0.42 | | 0.43 | | 0.88 | | 0.72 | | 10258 | | 6839 | 3.00 | | 2.98 | | | 0.72 | | 2.13 |
| **8** | F | CTRL | 16.0 | C | 24 | 0.44 | | 0.53 | | 0.84 | | 0.65 | | 9649 | | 5414 | 3.16 | | 2.59 | | | 0.75 | | 2.63 |
| **9** | M | CTRL | 12.0 | C | 41 | 0.55 | | 0.55 | | 0.86 | | 0.7 | | 8943 | | 7070 | 3.00 | | 2.61 | | | 1.22 | | 2.00 |
| **10** | M | CTRL | 12.0 | B | 20 | 0.65 | | 0.61 | | 1.05 | | 0.99 | | 7926 | | 5396 | **5.04** | | 4.05 | | | 0.83 | | 1.69 |
| **11** | M | CTRL | 19.0 | C | 44 | 0.49 | | 0.44 | | 0.84 | | 0.68 | | 8630 | | 5023 | 2.62 | | 2.46 | | | 1.52 | | **4.47** |
|  |  |  |  |  |  |  | |  | |  | |  | |  | |  |  | |  | | |  | |  |
| **All:** | 10 M, 1F | **Mean:** | 14.4 | 7C, 4B | 32.0 | 0.51 | | 0.51 | | 0.86 | | 0.73 | | 9329 | | 6310 | 2.89 | | 2.58 | | | 1.06 | | 2.51 |
|  |  | **SEM:** | 1.9 |  | 3.5 | 0.03 | | 0.03 | | 0.03 | | 0.04 | | 239 | | 233 | 0.27 | | 0.18 | | | 0.13 | | 0.25 |
| **Final:** | 7M, 1F | **Mean:** | 15.6 | 5C, 3B | 29.4 | 0.50 | | 0.51 | | 0.85 | | 0.72 | | 9646 | | 6629 | 2.77 | | 2.43 | | | 0.95 | | 2.33 |
|  |  | **SEM:** | 2.1 |  | 3.0 | 0.04 | | 0.04 | | 0.03 | | 0.05 | | 215 | | 203 | 0.17 | | 0.14 | | | 0.12 | | 0.17 |
|  |  |  |  |  |  |  | |  | |  | |  | |  | |  |  | |  | | |  | |  |
| ***Cocaine Abusers*** | |  |  |  |  |  |  | |  | |  | |  | |  | | |  | |  |  | |  | |
| **1** | M | COC | 10.5 | B | 30 | 0.49 | | 0.5 | | 0.85 | | 0.74 | | 8882 | | 6517 | 2.68 | | 2.38 | | | 1.25 | | 2.57 |
| **2** | M | COC | 13.0 | B | 33 | 0.2 | | 0.21 | | 0.45 | | 0.4 | | 9266 | | 5881 | 2.62 | | 2.84 | | | 1.37 | | 3.05 |
| **3** | M | COC | 12.0 | C | 39 | 0.31 | | 0.31 | | 0.73 | | 0.57 | | 9321 | | 6484 | 2.52 | | 2.34 | | | 1.11 | | 2.04 |
| **4** | M | COC | 18.0 | B | 32 | 0.32 | | 0.28 | | 0.9 | | 0.71 | | 11598 | | 7224 | 2.54 | | 2.34 | | | 0.63 | | 2.69 |
| **5** | M | COC | 11.5 | C | 40 | 0.5 | | 0.56 | | 0.97 | | 0.73 | | 11220 | | 6703 | 2.23 | | 3.51 | | | 0.69 | | 1.6 |
| **6** | M | COC | 14.0 | C | 34 | 0.48 | | 0.5 | | 0.79 | | 0.68 | | 10011 | | 6494 | 3.09 | | 3.38 | | | 0.87 | | 1.77 |
| **7** | M | COC | 12.0 | C | 41 | 0.48 | | 0.49 | | 0.96 | | 0.62 | | 10176 | | 6553 | 2.98 | | 3.16 | | | 0.88 | | 2.08 |
| **8** | M | COC | 8.0 | C | 23 | 0.61 | | 0.61 | | 1.07 | | 0.9 | | 10178 | | 6735 | 3.05 | | 2.95 | | | 0.64 | | 2.1 |
| **9** | F | COC | 12.5 | C | 39 | 0.69 | | 0.57 | | 0.83 | | 0.85 | | 9582 | | 7438 | 2.68 | | 2.83 | | | 0.76 | | **4.34** |
| **10** | M | **COC/ED** | 19.5 | C | 29 | 0.12 | | 0.12 | | 0.21 | | 0.21 | | 7298 | | 5502 | 2.27 | | 3.13 | | | 2.74 | | **4.98** |
|  |  |  |  |  |  |  | |  | |  | |  | |  | |  |  | |  | | |  | |  |
| **All:** | 9M, 1F | **Mean:** | 13.1 | 7C, 3B | 34.0 | 0.42 | | 0.42 | | 0.78 | | 0.64 | | 9753 | | 6553 | 2.67 | | 2.89 | | | 1.09 | | 2.72 |
|  |  | **SEM:** | 1.1 |  | 1.9 | 0.06 | | 0.06 | | 0.09 | | 0.07 | | 404 | | 188 | 0.10 | | 0.14 | | | 0.21 | | 0.37 |
| **Final:** | 8M | **Mean:** | 12.4 | 5C, 3B | 34.0 | 0.42 | | 0.43 | | 0.84 | | 0.67 | | 10082 | | 6574 | 2.71 | | 2.86 | | | 0.93 | | 2.24 |
|  |  | **SEM:** | 1.1 |  | 2.3 | 0.05 | | 0.05 | | 0.07 | | 0.06 | | 359 | | 140 | 0.11 | | 0.18 | | | 0.11 | | 0.19 |

Subjects shown in red were removed from the analysis of the final cohort (N=8 cocaine abusers; N=8 control subjects). Outliers are shown

highlighted in yellow. Values were derived from Microarray Analysis Suite version 5.0 analysis (available at [http://www.affymetrix.com](http://www.affymetrix.com/)). **Abbreviations**: COC, cocaine overdose; CTRL, control case; ED, excited delirium death; GAPDH, glyceraldehyde-3-phosphate dehydrogenase.
